# Factors Predicting Short Term Outcome in Children With Idiopathic Nephrotic Syndrome: A Prospective Cohort Study

**DOI:** 10.7759/cureus.21538

**Published:** 2022-01-24

**Authors:** Irshad Bajeer, Sabeeta Khatri, Seema Hashmi, Ali Lanewala

**Affiliations:** 1 Pediatric Nephrology, Sindh Institute of Urology and Transplantation, Karachi, PAK

**Keywords:** complete remission, relapsing illness, short term outcome, predictors, childhood nephrotic syndrome

## Abstract

Objective

The objective of the article is to determine the risk factors associated with relapses in children with idiopathic nephrotic syndrome (INS).

Material and methods

Fifty-seven children with the first episode of INS were included and followed up prospectively for a minimum period of one year to identify the risk factors related to relapses. The study subjects were divided into early (less than eight days) and late (equal to or more than eight days) responder groups and were compared in terms of the number of days to achieve complete remission, time to first relapse, and the pattern of relapse at the last follow-up.

Results

Of the 57 children, 32 (56%) were male and 25 (44%) female. The mean age of the study cohort was 5.3 ± 3 years. Sixteen (55%) children with ages ranging from one to four years had a higher propensity to develop relapse, although the p-value (p=0.11) was not significant. Gender analysis did not reveal any significant correlation (p=0.32); however, a higher proportion of males (n=17; 63%) responded within eight days of starting steroids than female counterparts (n=10; 37%). Microscopic hematuria at the disease onset was seen in 12 (21%) children, and out of them, five (41.6%) remained in complete remission. The mean time to achieve complete remission was 8.1 ± 3.5 days, while the early responder group had delayed time to first relapse as compared to the late responders (3.1 ± 5.2 vs. 1.6± 3.8; p=0.21). Among all the study participants, a significant number of children (n=20; 51%) were in complete remission at their last follow-up visit. Baseline serum albumin, cholesterol, body mass index (BMI), and serum creatinine had no significant difference.

Conclusion

The delayed response to steroids and younger age at presentation can predict the time to first relapse and number of relapses in children with INS, respectively.

## Introduction

Nephrotic syndrome is a common glomerular disease in children with an incidence of 1.2 to 16.9 per 100,000 children annually. It is idiopathic in 90-95% of cases, and 5-10% are secondary in etiology. The most common histopathological lesion is minimal change disease (MCD), followed by focal segmental glomerulosclerosis (FSGS) and mesangioproliferative glomerulonephritis (MesPGN). The mainstay of therapy for nephrotic children is corticosteroids, and according to the International Study of Kidney Disease in Children (ISKDC), approximately 90% of the children respond to this treatment, and out of them, 40-50% have a relapsing pattern of illness [1,2].

The risk of complications like hypertension, cataract, osteoporosis, obesity, growth retardation, infection, infertility, and behavioral disorders increase from the disease itself or because of immunosuppression. This relapsing disease with the potential to develop hazardous complications affects the child psychologically, and the family suffers economically, socially, and emotionally [3]. Multiple studies have tried to identify the risk factors associated with the relapsing pattern of illness, but it still remains unsolved how to predict these relapses and what should be the management strategy [4].

The age, gender, ethnicity, and initial time to respond to steroids are widely studied risk factors in the literature for predicting the outcome of children with idiopathic nephrotic syndrome (INS). The steroids take a median time of 11 days (IQR=10-15) to respond [5]. Constantinescu et al. predicted infrequently relapsing course in children achieving remission within a week of starting steroids [6]. Other studies showed that delayed response to steroids more than a week is associated with a frequently relapsing course, steroid dependency, and future steroid resistance [7].

The primary objective of this study is to find the risk factors associated with relapses between early and late responders to steroids. Both groups were compared in terms of time to first relapse, development of steroid-dependent nephrotic syndrome (SDNS), frequently relapsing nephrotic syndrome (FRNS), infrequently relapsing nephrotic syndrome (iFRNS), and secondary steroid-resistant nephrotic syndrome (SRNS). We have assessed the relationship of hematuria, age, and gender between both groups in our secondary outcome. None of the prospective studies, to our knowledge, have correlated outcomes of two cohorts of early and late responders to steroids.

## Materials and methods

This observational prospective cohort study was conducted at the Department of Pediatric Nephrology, Sindh Institute of Urology and Transplantation (SIUT) Karachi, Pakistan, from March to December 2019. The study was approved by the institutional ethics committee. Written informed consent was taken from the parents.

All the children aged from one to 12 years with the first episode of the nephrotic syndrome were enrolled in the study and followed up for a minimum period of one year. Children were included based on non-probability consecutive sampling. Exclusion criteria included the development of primary SRNS, follow-up less than 12 months, inability to understand the dipstick method for checking proteinuria, and refusal for consent. Follow-ups of children with relapsing illness were noted till the development of FRNS and SDNS. Children were divided into two cohorts based on time to achieve remission. Early responders achieved remission less than eight days, and late responders - equal to or more than eight days.

Definitions

Nephrotic syndrome is defined by proteinuria (urine albumin 3+ or 4+), hypoalbuminemia (serum albumin < 2.5 g/dL), high cholesterol (>200mg/dl) and edema [8]. A remission is achieved if the urinary protein is negative or trace in the first-morning sample for three consecutive days.** **Frequent relapses are two or more relapses in the six months of initial response or more than four relapses in any twelve months. Steroid dependence occur after two consecutive relapses when child is taking alternate day steroids or within two weeks of stopping steroids**​​​​​​​** [9].** **Steroid resistance occurs with the absence of remission despite therapy with daily prednisolone at a dosage of 2 mg/kg per day for four weeks [10].

Treatment protocol

According to current evidence-based management, the first episode of nephrotic syndrome was treated for 12 weeks. Initially, steroids were prescribed with a dose of 2 mg/kg daily for four weeks and, after achieving remission, switched to 1.5 mg/kg alternate day for eight weeks and then stopped without tapering. Those children who subsequently relapsed were given 2 mg/kg steroids for two weeks, then switched to 1.5 mg/kg alternate day for four weeks, and then stopped without tapering. Children with SDNS, FRNS, and secondary SRNS were treated with cyclophosphamide, levamisole, and cyclosporine, respectively as second line agents [11,12].

Statistical analysis

The quantitative variables were expressed as means with standard deviations and qualitative variables as percentages. Means were compared using the Student t-test. Categorical variables were assessed using the Chi-square test. The p-value <0.05 was considered as significant. All the data variables were analyzed using SPSS version 20 (IMB Inc., Armonk, USA).

## Results

During ten months, starting from March 2019 to the end of December 2019, fifty-seven newly diagnosed children with INS who fulfilled the inclusion criteria were enrolled in the study. Out of 57 children, 27 (47%) were early responders, and 30 (53%) were in the late responder group. The characteristics of both groups are shown in Table 1.

**Table 1:** Characteristics of early and late steroid responders BMI - body mass index; SDNS  - steroid-dependent nephrotic syndrome; FRNS - frequently relapsing nephrotic syndrome

	Early responders (n=27)	Late responders (n=30)	p- values
Boys n (%)	17 (63)	15 (50)	0.32
Age at onset in years (mean ± SD)	5.9 ± 3.1	4.8 ± 2.9	0.20
BMI in kg/m^2 ^(mean ± SD)	16.5 ± 1.7	17.6 ± 2.4	0.06
Microscopic hematuria n (%)	7 (26)	5 (16)	0.39
Serum albumin in g/dl (mean ± SD)	1.48 ± 0.44	1.59 ± 1.39	0.34
Serum cholesterol in mg/dl (mean ± SD)	375 ± 115	414 ± 122	0.21
Serum creatinine in mg/dl (mean ± SD)	0.25 ± 0.15	0.29 ± 0.17	0.42
Time for first relapse in months (mean ± SD)	3.1 ± 5.2	1.6 ± 3.8	0.21
No of relapses	1.19 ± 1.38	0.93 ± 1.11	0.45
Total follow up months	21 ± 5.1	17.8 ± 6.8	0.05
SDNS/FRNS	4 (15%)	3 (10%)	-

There was an equal distribution of males and females between both groups, and microscopic hematuria was slightly higher in early responders (26% vs. 16%; p=0.39). No significant results were found when we compared two groups in terms of the number of relapses and time to first relapse. However, the analysis showed that late responders had relapsed early (1.6±3.8 vs. 3.1±5.2 months; p=0.21), and they had less number of relapses than early responders (0.93±1.1 vs. 1.19±1.38; p=0.45). Likewise, insignificant results were seen between the two groups with respect to the age at the onset of disease, body mass index, serum albumin, serum creatinine, and serum cholesterol. Apart from the development of secondary SRNS in late responders, an unremarkable correlation was found between both groups in terms of outcome.

**Figure 1:**
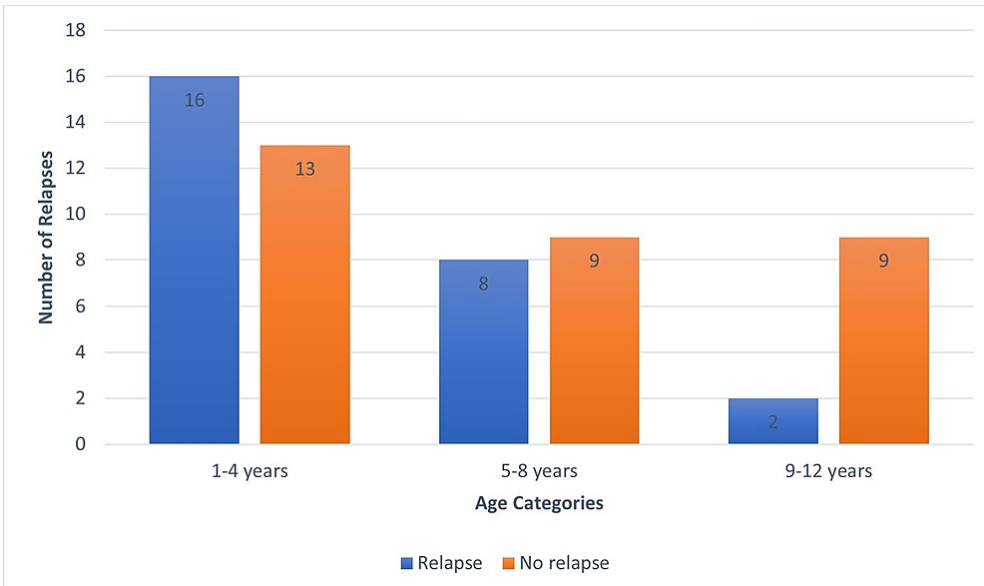
Relationship between different age groups and number of relapses

Children were divided into three age groups: 1-4 years (n=29; 51%), 5-8 years (n=17; 30%) and 9-12 years (n=11; 19%). The age distribution and its relationship with the number of relapses are shown in Figure 1.

The patients between ages one to four years were found to have a higher number of relapses as compared to others. However, this difference was not statistically significant (p=0.11). Children <5 years of age had a more relapsing illness; five (16.6%) developed SDNS/FRNS, and three (10%) developed secondary SRNS. Children >5 years of age showed relapsing diseases in two (7.4%) and did not develop secondary SRNS, though the p-value was not statistically significant (p=0.139).

In terms of the development of SDNS/FRNS, no significant correlation (p=0.577) was found between males (n=5; 15%) and females (n=2; 8%). The majority of the children with no evidence of microscopic hematuria achieved sustained remission (n=24; 54%), while the rest of them developed infrequent INS (n=14; 31%), SDNS/FRNS (n=5; 11%) and secondary SRNS (n=2; 4%).

**Figure 2:**
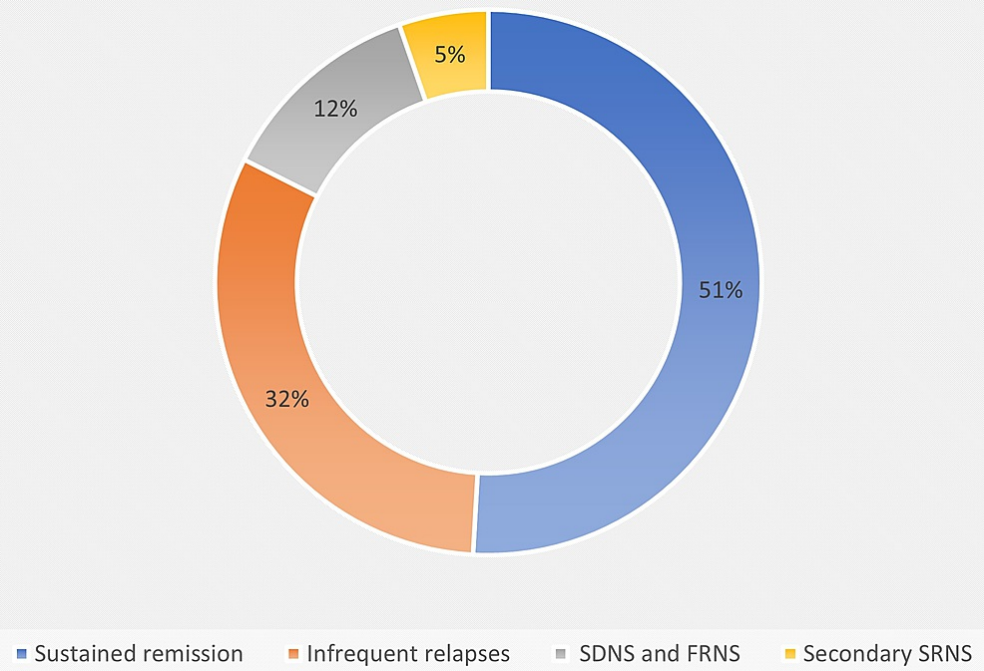
Final outcomes of patients SDNS - steroid-dependent nephrotic syndrome; FRNS - frequently relapsing nephrotic syndrome; SRNS - steroid-resistant nephrotic syndrome

Among the 57 included children, 29 (51%) remained in sustained remission, 18 (32%) had infrequent INS, seven (12%) showed SDNS or FRNS, and three (5%) developed secondary SRNS (Figure 2).

Three children with secondary SRNS had minimal change disease on renal histology. The study participants had a mean follow-up of 19.3 ± 6.2 months. A total of 47 (82%) children responded within 10 days of initiation of steroids, and out of them, 38 (81%) had an infrequently relapsing course at the last follow-up.

## Discussion

The long-term outcomes of children with steroid-sensitive nephrotic syndrome are far better than a resistant disease. A sub-group of SSNS that behaves as frequently relapsing or steroid-dependent nephrotic syndrome are subjected to recurrent steroid therapy, rendering them to fatal side effects.

The majority of studied children were between one to four years of age. Moreover, when it comes to the age at onset of disease and frequency of relapses, an inverse relationship was found, where the number of relapses decreased with the advancing age. This observation is identical to the outcomes reported by Bhatta M et al., Mishra et al., Kabuki et al. [13-15]. On the other hand, Shuichiro et al. and Takeda et al. didn't find any significant correlation between age and the number of relapses among children with INS [16,17].

In our study sex ratio was about 1.28:1, which is lower than previously reported [6,13]. In contrast to females (n=9; 36%), more relapses were found in males (n=7; 53%); similar findings were reported by Noer et al. [18]. In terms of time to achieve remission majority of males (n=17; 63%) responded within eight days of starting steroids than females (n=10; 37%). Sureshkumar et al. had similar results among Australian children with INS [19].

The majority of studied children achieved sustained remission (n=29; 51%), while only a small proportion of patients developed SDND/FRNS (n=7; 12.3%). Almost similar results were reported by Mishra et al., in which 41% of children achieved complete remission and 8% developed the relapsing disease during the first year of treatment completion [14]. The early response to steroids predicts the ultimate outcome of childhood nephrotic syndrome. Children in our study achieved complete remission within 8.1 ± 3.5 days, which is similar to research conducted by Yap et al. [20]. In contrast to these results, Constantinescu et al. reported 13.9 days to achieve remission [6]. Our data showed that the time to first relapse is 3.1 ± 5.2 months in early responders while 1.6± 3.8 in late; however, the duration in early responders is almost double, but it is not statistically significant (p=0.21).

ISKDC has reported that a number of relapses in the initial period is highly predictive of outcome in subsequent years [21]. With respect to our study, relapsing illness was mainly observed in early responders in comparison to late responders (n=4; 15%). However, other studies exhibited opposite results. For instance, the study by Carter et al. showed more relapsing disease among late responders, where the duration to attain initial remission with steroids was greater than nine days [7]. Similar results were reported by Nakanishi et al., Harambat et al., and Vivarelli et al. [22-24].

The incidence rate of relapse was 54.4 in 1000 child follow-up months in 57 children, which is higher than 42.6 reported by Gebrehiwot et al. [3]. At presentation, the hematuria was observed in 12 (21%) patients, and the reported incidence of microscopic hematuria is around 30% which is slightly higher than our cohort. We didn't find any correlation of hematuria with the pattern of relapses and time to achieve remission. A high cholesterol level at presentation had no effect on relapse in our patients, and similar results were published by Mishra et al. [13].

## Conclusions

The early response to steroids could predict future relapses and the late responders had a lower number of relapses which remains unexplained. Children younger than five years may have higher chances of developing SDNS and FRNS. Physicians should be vigilant to monitor these patients closely and counsel the families of nephrotic children regarding the prediction of subsequent relapses and ultimate outcome.
